# Evolved *Fusarium oxysporum* laccase expressed in *Saccharomyces cerevisiae*

**DOI:** 10.1038/s41598-020-60204-1

**Published:** 2020-02-24

**Authors:** Natalia Kwiatos, Marzena Jędrzejczak-Krzepkowska, Agnieszka Krzemińska, Azar Delavari, Piotr Paneth, Stanisław Bielecki

**Affiliations:** 1 Institute of Technical Biochemistry, Faculty of Biotechnology and Food Sciences, Lodz University of Technology, Stefanowskiego 4/10, 90-924, Lodz, Poland; 2 Institute of Physics, Lodz University of Technology, Wólczańska 219, 90-924, Lodz, Poland; 3 Independent scholar, Tehran, Iran; 4 Institute of Applied Radiation Chemistry, Faculty of Chemistry, Lodz University of Technology, Wróblewskiego 15, 93-590, Lodz, Poland

**Keywords:** Metalloproteins, Oxidoreductases, Biocatalysis, Enzymes, Proteins, Structural biology, Molecular modelling, Protein design, Environmental biotechnology, Expression systems, Molecular engineering, Protein analysis, Protein design, Biochemistry, Biotechnology, Computational biology and bioinformatics, Structural biology, Molecular modelling

## Abstract

*Fusarium oxysporum* laccase was functionally expressed in *Saccharomyces cerevisiae* and engineered towards higher expression levels and higher reactivity towards 2,6-dimethoxyphenol, that could be used as a mediator for lignin modification. A combination of classical culture optimization and protein engineering led to around 30 times higher activity in the culture supernatant. The winner mutant exhibited three times lower Km, four times higher kcat and ten times higher catalytic efficiency than the parental enzyme. The strategy for laccase engineering was composed of a combination of random methods with a rational approach based on QM/MM MD studies of the enzyme complex with 2,6-dimethoxyphenol. Laccase mediator system with 2,6-dimethoxyphenol caused fulvic acids release from biosolubilized coal.

## Introduction

Laccases are oxidoreductases that catalyze the 4-electron reduction of O_2_ to water with simultaneous oxidation of organic substrates. Laccases are able to catalyze oxidation of a substrate of interest directly or indirectly by the formation of a radical, which then may take part in a non-enzymatic event that effects in the oxidation of a substrate of interest. Laccases oxidize phenolic substrates but are also able to oxidize non-phenolic or bigger substrates by Laccase Mediator System (LMS), where a small phenolic compound acts as a mediator^1^. Laccases contain 4 copper atoms buried in their 3D structure, which are located in two separate Cu centers. T1 copper ion is a mononuclear center, whereas T2 copper ion and two T3 copper ions are positioned in T2/T3 copper center. The substrate is oxidized closed to T1 copper ion and then the electrons are transferred to the tri-nuclear center where O_2_ is reduced to water. The coppers are coordinated by nearby located residues: the T1 copper ion by one Cys and two His residues, the T2 copper by two His residues and a solvent molecule and T3 copper to three His residues^2,3^.

Biosolubilization of brown coal is a clean coal technology that aims at the conversion of the lignite to its cleaner form or to change its structure to gain new features^4,5^. Such solubilized material could be used as a source of value-added products^4^. The liquid form of coal – microorganisms, such as *Fusarium oxysporum* reported before as an excellent coal solubilizer^6^, uses their metabolites, alkaline substances, biosurfactants and enzymes to turn the solid polymer to black liquid. Laccases, from bacteria and fungi, for example from species *Pleurotus*^7^ or *Streptomyces*^8^, are known to take part in the degradation of lignin and lignite. These two polymers are similar in structure, thus, the mechanism of their degradation is expected to be the same. Lignin comprises only 10–20% of phenolic subunits. In theory, laccase could act on those subunits present on the lignin surface and modify the polymer. However, the possibility of lignin subunits entering the laccase active site is very limited. Moreover, it is not known to what extent lignin could be modified in this way^9^. According to literature data, the treatment of lignin with sole laccase leads to polymerization instead of depolymerization^9,10^. However, the treatment of lignin with LMS could lead the reaction in both directions depending on reaction conditions, the polymer, and the products of the first cycles of reactions^9^. Nevertheless, the structure of lignin promotes polymerization. The higher the redox potential of a substrate the more probable it is that laccase tends to polymerize instead of depolymerize. Due to the abundance of many phenolic subunits of lignin, polymerization may be an energetically favorable event. The degradation of lignin by LMS is still a challenge.

In this article, we report *F. oxysporum* Gr2 laccase functional expression in *Saccharomyces cerevisiae* and its engineering. The substrate scope of this enzyme was studied with docking and molecular simulations techniques. *S. cerevisiae* strain enables easy genetic manipulations and convenient protocol for directed evolution of expressed proteins. These features were used in laccase mutagenesis towards higher activity towards 2,6-dimethoxyphenol (DMP) which can act as laccase mediator in brown coal degradation. The winner mutant was purified and characterized. Moreover, the impact of the laccase variants on solubilized coal was assessed.

## Results and Discussion

### Molecular simulation studies

**Figure 1:**
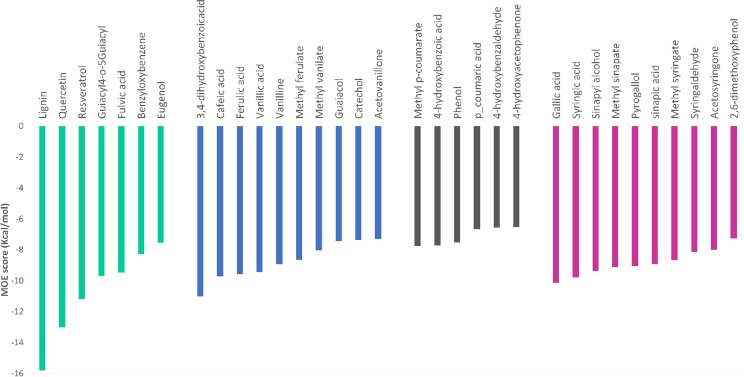
Docking results. Purple – syringyl derivatives of lignin, grey – p-hydroxyphenol derivatives of lignin, blue – guaiacyl derivatives of lignin, green – other potential laccase substrates.

Molecular simulation studies aimed to find a suitable substrate for Gr2 laccase. The substrate would play the role of a mediator in the LMS for brown coal biosolubilization. Homology models were used in docking studies previously with success^11–14^. To our knowledge, there were only two studies concerning laccase docking with multiple substrates^11,13^. The authors of the first one aimed to study laccases from *Yersinia enerocolitica* in terms of their affinity to 10 different substrates and proved that guaiacol, lignin monomers and ABTS are, among others, true substrates of the enzyme^13^. Another study characterized isoenzymes of *Ganoderma* sp. laccase and revealed that the enzymes have a higher affinity to ABTS than to lignin derived phenols^11^. In our study, 33 docked complexes were obtained and five of them were further analyzed. The substrates were chosen on the bases of literature search^15–18^. Guaiacol, *p*-coumaric acid, sinapic acid (SA), fulvic acid and DMP were selected according to the best MOE docking score (Supplemental Table S1), economic and feasibility analysis of the use of such a compound in LMS (Fig. 1).

**Figure 2:**
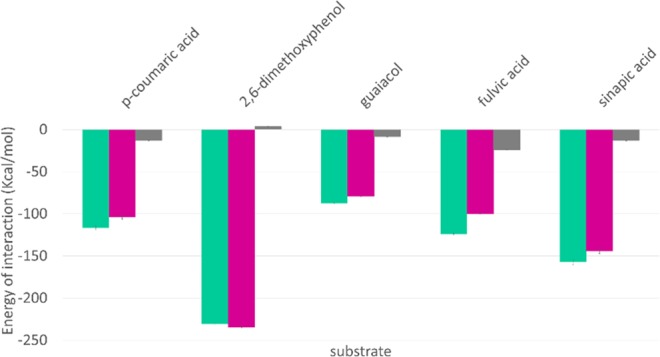
The interaction energy results. Grey - van der Waals energy, purple - electrostatics, green - summary energy of interaction.

In the next steps, obtained complexes were subjected to 1 ns of QM/MM MD simulations in order to study protein-ligand interactions. The energies of interactions were calculated for over 1000 structures for each complex. Figure 2 shows the summary energy of interactions for each enzyme-substrate pair and demonstrates if the energies are due to electrostatic or van der Waals interactions. The lowest energy was observed for DMP, a syringyl lignin derivative, which means the highest affinity of the enzyme to these substrates. Syringyl derivatives possess two methoxy groups on a phenol ring and this may be the reason for the lowest energy of interaction. Methoxy groups may cause stronger interaction between the residues in the binding site and themselves, which is visible in the low energy of interaction calculated for the residues closest to the ligand.

**Figure 3:**
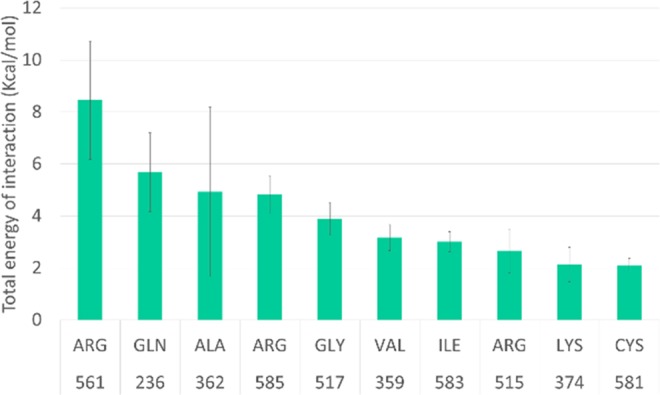
First ten residues with the highest energy of activation.

The complex of laccase with DMP was subjected to deeper studies of protein-ligand interactions. As a result, a list of the energy of interactions of each laccase residue with the substrate was obtained. Figure 3 presents the first 10 residues with the highest energy – residues that may disturb substrate binding. These residues were treated as hot spots for enzyme evolution.

### Expression and engineering of laccase

The vector for expression of laccase in *S. cerevisiae* was constructed successfully and the level of expression was checked in 96-well plate format. The average activity of laccase after the cultivation of recombinant *S. cerevisiae* on MEM medium was 20 U/L (ABTS assay, pH 3).

The laccase was subjected to protein engineering in order to enhance its expression and its activity towards a redox mediator (DMP) that would improve brown coal modification by the enzyme. pH 5 was chosen as a condition for screening, as most processes of bioconversion of lignocellulose prefer pH 5 and the new laccase could find then application not only in brown coal biodegradation but also in biorefinery development^18–21^.

**Figure 4:**
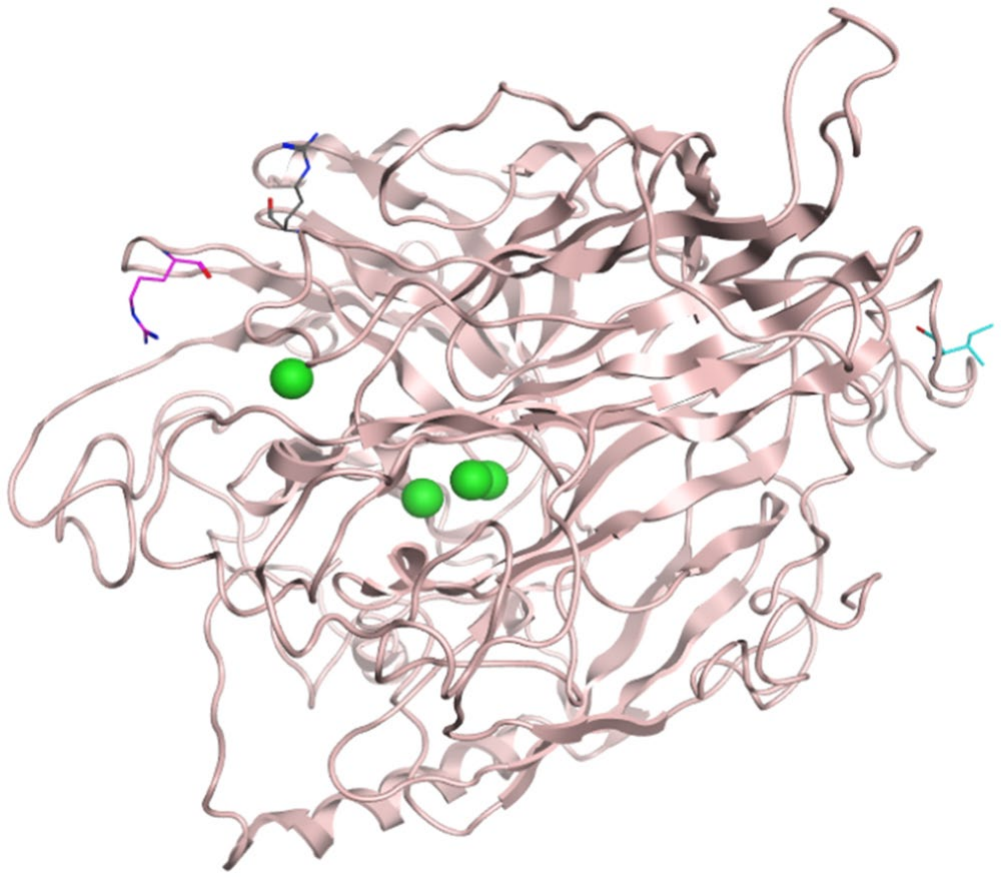
Position of mutated residues on Gr2 laccase model. Cyan sticks– Ile52, pink sticks Arg515, grey sticks– Arg561, green spheres – copper atoms.

**Figure 5:**
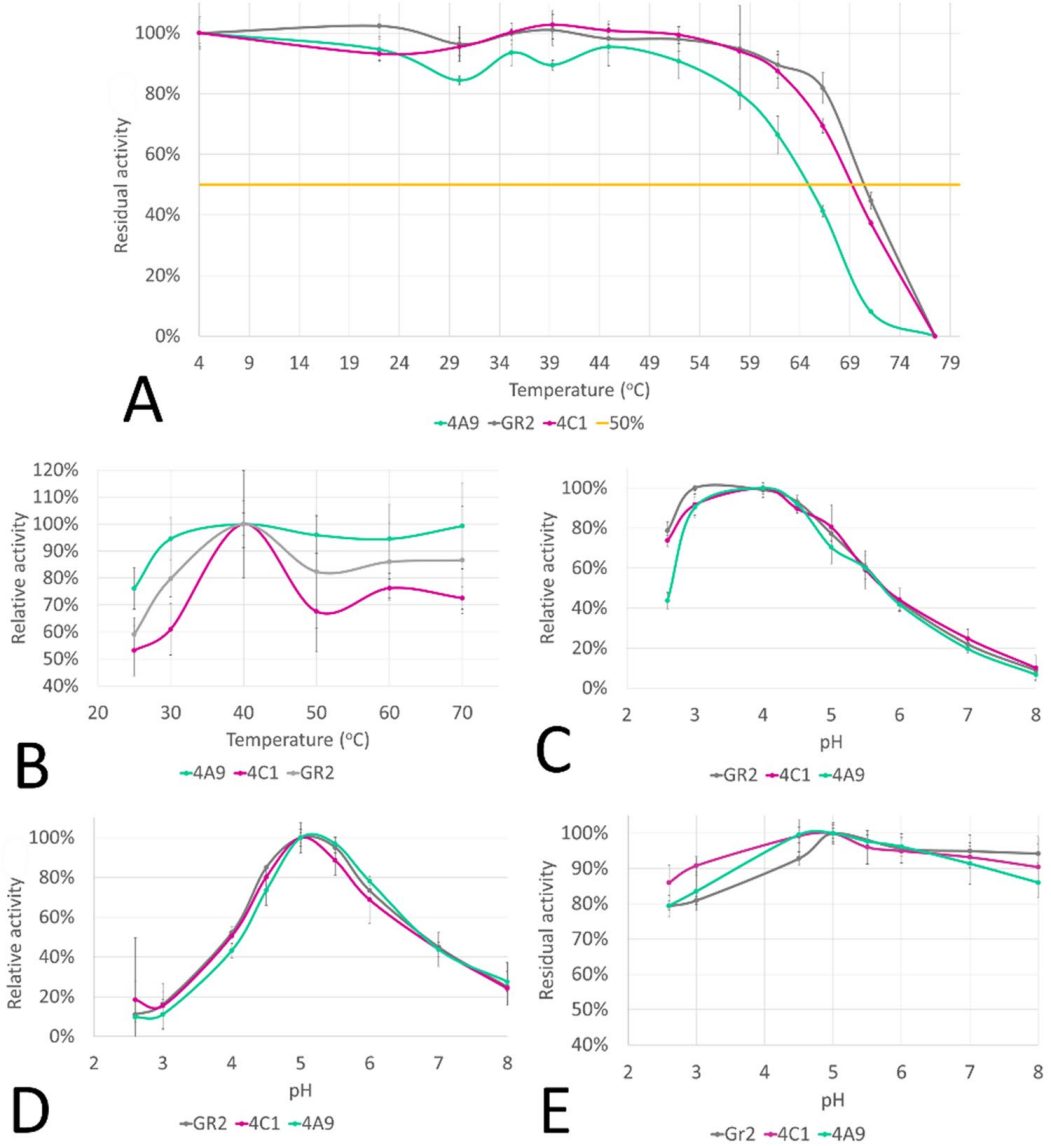
Characterization of 4A9 laccase mutant variant: (**A**) thermostability profiles, (**B**) activity in temperature, (**C**) activity in pH for ABTS, (**D**) activity in pH for DMP, (**E**) stability in pH.

The successful winner of the first round of evolution (error-prone PCR), 4C1 mutant, contained Ile at position 52 instead of Thr and it exhibited 6 times higher activity. The similar increase in activity towards both substrates suggests that the reason for such success lies in the more effective expression of the protein. The amino acid lies on the surface of the protein (Fig. 4), far from the catalytic center of the enzyme and co-creates a loop. This mutation may cause the formation of a new hydrogen bond between Gly51 and Thr52. Mutations on the surface of an enzyme have been discussed before as those that may influence the stability of the enzyme. For example, in the study of *P. cinnabarinus* laccase, a mutation that was introduced on the surface of the protein is claimed to enhance the flexibility of the enzyme, as the existing hydrogen bonds were broken. The authors theorized that these may have facilitated the folding of the protein in the posttranslational phases. Another mutation on that enzyme’s surface caused the formation of new hydrogen bonds. Both of them contributed to enhancing the activity of this laccase^22^. Another example is the mutagenesis of Ala to Thr at position 361 in high redox potential laccase from basidiomycete PM1. This mutation caused the formation of an additional hydrogen bond with Ser372 and according to the authors strengthen the rigidity of the enzyme^23^. If we compare the thermostability of Gr2 laccase with 4C1 mutant (Fig. 5A), we may notice that there is not much difference. Although Ile52Thr mutation did not increase the stability, this feature was not significantly disturbed, which is often the case in directed evolution studies^24^.

**Figure 6:**
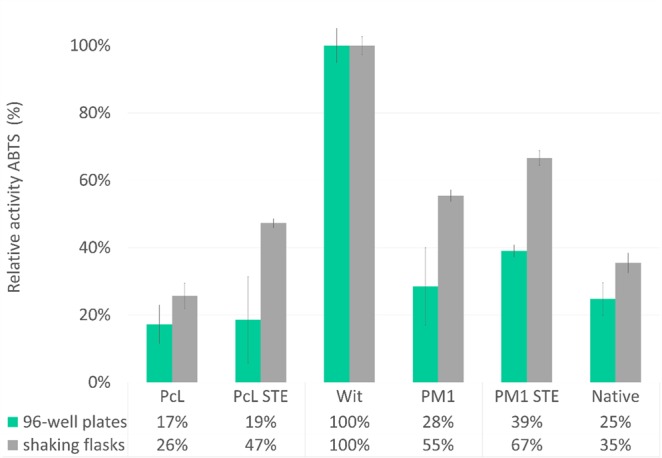
Laccase expression with evolved alpha factors in 96-well plates (green) and in shaking flasks (grey). The highest activity in each experiment was set to 100%.

It was shown before that the evolution of peptide sequences enhances the expression of proteins by improvement in secretion. It was believed that each protein should have its own designed signal peptide, as the mutations are somehow correlated with the target sequence. In order to use the evolved peptide for another protein, the sequences should be very similar^25^. Pioneering work of Wittrup’s group^26^ suggested that evolved signal peptides could be used for the expression of various groups of proteins. Hence, it was decided to use three alpha factor pre-pro leader that were evolved and described before^25,26^ in order to enhance the secretory levels of 4C1 laccase mutant. Wit signal peptide led to the highest expression level in our experiment (Fig. 6). The results are surprising due to the fact that originally, this signal peptide was evolved for an antibody.

Alpha factor pre-pro leader has three sites for cleavage by KEX2, KEX1 and STE13 proteases. In the case of overexpressed proteins, there is a concern of the inefficient processing of peptide by STE13 protease. The amount of the protease may be insufficient in comparison to the amount of expressed enzyme. As a consequence, it was observed before that the expressed protein obtain an extra EAEA peptide on its N-termini, which in many cases may interfere with the activity of the target enzyme^24^. That is why the PM1 and Pcl signal peptides were also designed without STE13 cleavage site and tried for the enhanced secretion of 4C1 mutant. According to our results secretion of Gr2 laccase is better for PM1 and Pcl signal peptide retaining the STE13 site. However, the correlation may change with higher expression levels obtained for the laccase. Interestingly, Wit signal peptide does not obtain the STE13 site at all and the results are much better than the other peptides. The alpha factor evolved by Wittrup’s group was used in further study.

The improvement of the activity towards the small compound would indirectly improve brown coal modification. Gr2 laccase can catalyze the oxidation of DMP and then oxidized DMP can oxidize lignite leading to its modification. The functional hot spots given by HotSpotWizard 2.0 (Supplemental Table S2) were compared to QM/MMMD results. Two residues indicated by both methods were chosen for combinatorial mutagenesis studies Arg515 and Arg561. The 4A9 mutant was selected as the winner of the third generation of mutagenesis. The mutant exhibited 30% higher activity towards DMP and no change of activity towards ABTS. At both positions 515 and 561 Arg was exchanged with Thr. Arg561 and Arg515 are situated on the surface of the protein (Fig. 4), in the entrance to the substrate binding site. Exchange of bulky amino acids to smaller ones was described before as a method for widening substrate binding site, thus expanding the substrate scope of the enzyme^27,28^. Such an approach could be beneficial for lignite modification. During the process, small phenolic substances are being created and they could act as additional laccase mediators and in this way improve the efficiency of the process. That is why Arg515 and Arg561 were chosen as the hot spot for combinatorial saturation mutagenesis by *in vivo* overlap extension (IVOE)^29,30^. The mutant with the highest activity was the one with a mutation to Thr at both positions. The mutation to smaller amino acid causes a change in the architecture of the area close to the entrance to the active site. Arg with long electrically charged side chains occupies more space than Thr which may disturb some substrates to enter the binding pocket (Fig. 4). The distance between Ser235 (another amino acid in the vicinity of binding site entrance) and Thr515 is longer than the distance between Ser235 to Arg515 (11.3 and 7.9 Å respectively), which caused the affinity to be increased towards DMP (Table 1). Other studies described the mutation of an amino acid residue to a smaller one. For example, Tyr193 was mutated into Cys in *Enterobacter cloacae* esterase, which caused a decrease in the steric hindrance near the enzyme binding pocket. As a result the substrate and product could enter and exit the active site more freely^31^. In the study of *P. chrysosporium* alcohol oxidase, one of mutation of Phe313 to Leu caused a small increase in space in the cavity and the enzyme gained the possibility of converting glycerol^28^.

**Table 1:** Kinetic constants for Gr2 laccase and its mutants towards ABTS and DMP.

Variant	Km (µM)	Specific activity (U/mg)	kcat (s^−1^)	kcat/Km (µM^−1^*s^−1^)
**ABTS**				
Gr2	721.8	6.23	7.48	0.01
4C1	48.5	8.50	10.27	0.21
4A9	231.6	35.4	42.71	0.18
**DMP**				
Gr2	739.0	10.28	12.34	0.02
4C1	504.4	11.55	13.96	0.03
4A9	271.7	49.97	60.29	0.22

### Production, purification and characterization of 4A9 laccase variant

The 4A9 mutant was produced in five 2 L shaking flasks and the maximum level of expression was around 600 U/L. The yield of purification was 25% where 35 U/mg specific activity was reached (Supplemental Table S4, Supplemental Figs. S1 and S2).

The 4A9 mutant had optimal activity at 40 °C (Fig. 5B). 4A9 mutant gained higher activity in both lower and higher temperatures compared to the native enzyme. In 25 °C, the activity of Gr2 was below 60%, while the activity of 4A9 mutant was 16% higher. Moreover, the activity of the final mutant 4A9 in higher temperatures was also enhanced. Most fungal laccases have their optimal activity at higher temperatures. For example, laccase from *Coprinopsis cinerea* (basidiomycetes) at 60 °C^32^, *Pycnoporus sanguineus* at 65 °C^33^ and *Melanocarpus albomyces* (ascomycetes) at 60–70 °C^34^ have their optimal activity. The thermostability of the enzymes is comparable to other laccases expressed in *S. cerevisiae* (T_50_ around 70 °C): *P. cinabarinus* laccase, PM1 laccase or *T. versicolor* laccase^22,35,36^ (Fig. 5A).

The activity of Gr2 laccase and its mutants was strongly related to pH. In the case of ABTS substrate, Gr2 laccase is most active at pH 3, while the pH optimum for 4C1 and 4A9 mutants is around pH 4 (Fig. 5C). The activity towards DMP for all variants retained 90% activity in the range of pH between 3 and 4.5 (Fig. 5D). Gr2 laccase and its mutants were most stable at pH close to 5 (Fig. 5E). All three variants exhibit considerable and similar stability towards storage at different pH.

Kinetic constants for Gr2 laccase and its mutants are given in Table 1. The table clearly shows the progress of protein engineering – 4C1 mutant is already an improved variant in terms of Km and catalytic efficiency and 4A9 mutant, the winner of the whole campaign has the lowest Km, the highest kcat and kcat/Km. The Km was almost three times lower for 4A9 mutant than the Km of the parental enzyme. Although, the biochemical tests for Gr2 and 4C1 mutant were performed on unpurified samples and the specific activities might be unreal, the trend of catalytic improvement was kept. If one compares the kcat and kcat/Km for all three unpurified samples, it can be noticed that both values are the highest for 4A9 mutant (not shown in the table; unpurified 4A9 exhibited kcat of 48 s^−1^ and catalytic efficiency of 0.2 µM^−1^s^−1^). The Km of many fungal laccases is in the range of 20–30 µM^36–38^, which is 10 times lower than for 4A9 mutant. The laccase from *Thielavia* sp. has the turnover for DMP 3.46 s^−1^ and catalytic efficiency of 0.17 µM^−1^s^−1^, whereas, the laccase from another Ascomycota *B. aclada* has the kcat of 45.6 s^−1^ and kcat/Km of 2.4 µM^−1^s^−1^. Hence, Gr2 laccase and its variants had quite high turnover numbers, however, the overall catalytic efficiency could be improved. Future protein engineering studies could focus on enhancing the affinity of the enzyme to the substrate.

### Brown coal modification

**Figure 7:**
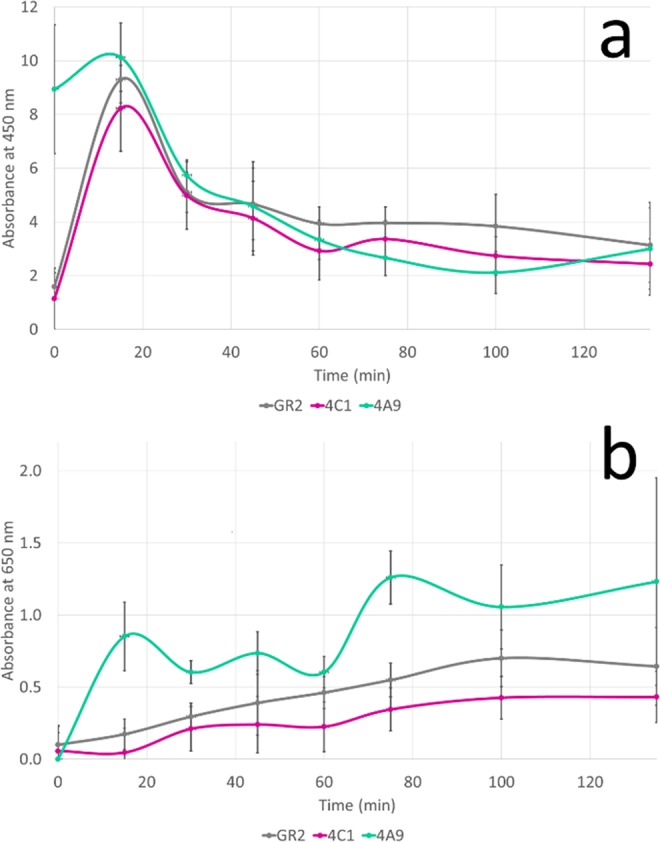
Changes in humic acids (**a**) and fulvic acids (**b**) presence during process of brown coal modification by laccase variants.

Gr2 laccase is able to degrade and modify solubilized brown coal (Fig. 7) in LMS. The docking studies confirmed by biochemical studies indicated DMP to be used as a mediator for this process. In the first minutes of the process, humic acids were formed which is illustrated by an increase in absorbance at 450 nm (Fig. 7a). Then, the number of humic acids decreased, which could be caused by their degradation to fulvic acids (simultaneous increase in absorbance at 650 nm) (Fig. 7b). 4A9 mutant produced the highest amount of fulvic acids during the same unit time as Gr2 and 4C1 laccases. Part of the products created in the process with 4A9 mutant might have become substrates for further conversions and that is why we observe an undulating curve in Fig. 7b for the increase in fulvic acids amount. Other variants are less effective and this is probably the reason for the difference in the shape of the curves. Table 2 presents the increase of fulvic acids release for all variants. It indicates that 4A9 mutant is the most effective one – it doubles the production of fulvic acids in comparison to Gr2 laccase.

**Table 2:** Increase in fulvic acid release during the process of brown coal modification in presence of 2,6-dimethoxyphenol in regard to the control – solubilized coal with the mediator.

Variant	Gr2	4C1 mutant	4A9 mutant
Increase in fulvic acids release during the process	29%	17%	54%

## Conclusions

In this study, *F. oxysporum* Gr2 laccase was expressed for the first time in *S. cerevisiae*. The relatively low expression level was enhanced around 30 times due to a combination of protein engineering and classical culture optimization. A new enzyme was designed by random mutagenesis and site-saturation techniques supported by molecular simulation studies. The new laccase carries three mutations – Ile52Thr, Arg515Thr and Arg561Thr, which contributed to 10 times higher catalytic efficiency of the enzyme. The 4A9 laccase mutant is most active at 40 °C and loses only up to 20% activity when the reaction takes place either in higher or lower temperatures. The mutagenesis did not disturb the stability of the protein. All the above features make the mutant a very promising tool for potential industrial applications, such as brown coal solubilization of lignin modification. The efficient laccase mediator system with DMP was proposed. This paper demonstrates the first such advanced study concerning usage of laccase for lignite modification.

## Materials and Methods

### Materials

*S. cerevisiae* BJ5465 strain (ATCC 208289) used in this study was obtained thanks to the courtesy of Dr Miguel Alcalde, CSIC, Madrid. All chemicals used in the study were analytical grade and bought from Sigma-Aldrich or Chempur. The brown coal used in the study was obtained from Bełchatów Brown Coal Mine (Poland). Coal particles with a diameter of 1–2 mm were subjected to pretreatment with ozonation and then subjected to microbial solubilization as explained before^5,39^.

### Methods

#### In silico studies

Preparation of Gr2 laccase model for molecular dynamics: The backbone of Gr2 laccase was made of 640 amino acids with four copper ions coordinated in an optimal position to stabilize second-order protein structure. The parameter set for the copper atoms is included in Supplemental Table S5-Table S7. Cu–L distances and L–Cu–L angles, were taken from the available DFT-optimized model structure described by Comba and Remenyi^40^. To maintain interactions between copper atoms and neighboring residues constraint conditions have been applied with force constant 2500 kJ/(mol*A^2^). Force field parameters and electrostatic charges of atoms for protein were derived from the AMBER libraries [FF99SB force field]^41^. To confirm the protonation states of amino acids, especially His residues, the pKa values were computed by using PROPKA3.0^42^ software at pH = 7.0. Hydrogen atoms were added using the tLEAP module of AMBER program^43^. The system was neutralized by the addition of 30 sodium cations in optimal positions indicated by AM1 calculations implemented in tLEAP. The system was soaked in the orthorhombic box of TIP3P water molecules of the size 120×128×120 Å. Afterward, energy minimization was applied to relax the whole structure by using the AMBER11 program^44^.

Molecular docking: The molecular docking studies were performed by using the MOE docking program^45^. The whole set of protein binding pocket residues were chosen for the docking site and treated flexible upon docking. The structures of 33 ligands were prepared and optimized with the MMFF94 force field^46^. The 30 docking runs were estimated to be efficient enough to list the possible poses. The London ΔG scoring function was used to rank order of the different poses generated. Based on the obtained results, ligands: coumaric acid, DMP, fulvic acid, guaiacol, and SA were selected for further investigation and molecular dynamics studies.

Molecular dynamic studies: Parameters for the five ligands chosen were obtained using GAFF^47^ from the tLEAP module. Subsequently, several optimizations and dynamic simulations for each ligand-protein complexes were performed using AMBER ff99SB^48^ force filed of AMBER11 with a time step of 1 fs. Periodic boundary conditions, the particle mesh Ewald method^49^ and the *cut-off* for nonbonding interactions with a range radius from 14.5 to 16 Å were applied. The systems were heated from 0 to 300 K with 0.001 K step size. Equilibration of complexes was achieved during 500 ps of Langevin-Verlet dynamics at 300 K. Complexes were stabilized by performing 1 ns of NVT MM MD. In order to study protein-ligand interactions, the 1 ns of QM/MM molecular dynamics simulations were performed using AMBER11, where the QM part, only ligands at this stage, was treated at the PM3 semiempirical level of theory^41^. VMD^50^ and DiscoveryStudio ver.3.5^51^ were used for visualization of complexes.

Analysis of substrate-enzyme interactions: To study interactions between ligands and protein residues the free energy decomposition per-residue scheme has been applied as implemented in AMBER11 by using MMPBSA module^52^. MMPBSA is a post-processing method that requires trajectories obtained during QM/MM molecular dynamics simulations (.mdcrd file with trajectory) and allows to study specific interactions between ligand and protein residues. Hence, previously obtained trajectories from five selected ligands simulated during 1 ns PM3/AMBER studies were used for further per-residue interaction analysis. Afterward, one of them was selected as the most promising structure and additional PM3/AMBER dynamics simulation was performed, with the QM part composed of the substrate, Asp293, Cys363, one copper cation, and three water molecules. Finally, the interaction analysis for the substrate treated more precisely with the bigger QM part was computed.

Vector construction and expression of Gr2 laccase: The coding sequence of Gr2 laccase (GeneBank accession number MH351668) was subjected to two PCR reactions. The first one amplified laccase coding sequence producing 40 nucleotide long overhangs - on the 5′ end homologous to α factor sequence and on the 3′ end homologous to pYES2 vector. The second reaction amplified α factor sequence producing overhangs – on the 5′ end homologous to pYES2 vector and on the 3′ end homologous to the laccase coding sequence. The primers and PCR conditions used are given in Supplemental Table S8. Three DNA sequences were prepared for transformation to competent *S. cerevisiae* cells: linearized with *Sac*I and *Not*I purified from agarose gel pYES2 vector, α factor sequence and the laccase coding sequence. The sequences, 100 ng each, were co-transformed to *S. cerevisiae* cells according to Yeast Transformation Kit (Sigma-Aldrich). The cells were grown on selective SC dropout plates for 3 days at 30 °C at stationary conditions. We used SC medium for the cultivation of *S. cerevisiae* (1.92 g/l yeast synthetic dropout-medium supplement without uracil, 6.7 g/l yeast nitrogen base without amino acids, 2 g/l glucose or raffinose, 25 µg/ml chloramphenicol), YPD for the cultivation of selected clones in rescreening step (2% peptone from casein, 1% yeast extract, 0.4% glucose, 25 µg/ml chloramphenicol) and laccase minimal expression medium (MEM) with 0.2 mM CuSO_4_ (1.92 g/l yeast synthetic dropout-medium supplement without uracil, 6.7 g/l yeast nitrogen base without amino acids, 2 g/l galactose, 25 µg/ml chloramphenicol)^24^. The medium was inoculated by transferring a single yeast colony by a sterile toothpick. Wild-type *S. cerevisiae* was cultivated in the same media but with the addition of 20 mg/L uracil. Plates were sealed and put in a box with wet paper towels to prevent evaporation and cultivated for 3 days at 30 °C and 250 rpm. Then the plates were centrifuged for 15 min at 1149 × g, 4 °C.

1^st^ round – Error-Prone PCR: The Gr2 coding sequence was subjected to PCR with *Taq* polymerase with a previously set 25 µM MnCl_2_ end concentration (Supplemental Table S9 and S10). 100 ng of linearized pYES2 and 150 ng of the PCR product were co-transformed to *S. cerevisiae* competent cells. The colonies that grew on the selective SC dropout plates were picked and cultivated on 96-well plates in MEM for 72 h, 30 °C and 250 rpm. Next, the screening was performed in order to choose the winner for the next round of enzyme evolution. Individual clones were selected and cultured on 96-well plates in expression medium as described above. In each plate, the 6^th^ column was inoculated with *S. cerevisiae* cells carrying a pYES2 plasmid with a parental type gene, while H1 well was inoculated with wild-type *S. cerevisiae*. The cultivation took place as explained above. Next, the plates were centrifuged (1149 × g, 15 min, 4 °C) and 20 µl supernatant was transferred from master plates to two fresh 96-well replica plates by pipetting machine (Hamilton). The screening of mutant libraries consisted of two assays – with ABTS or DMP. The activity was measured at 25 °C by monitoring the oxidation of 5 mM ABTS (ε_420_ = 36,000 M^−1^cm^−1^) or 8 mM DMP ε_469_ = 49,600 M^−1^cm^−1^) in McIlvaine buffer of pH 5. Next, the best mutants were subjected to first, second and third re-screening as described earlier^22^, in order to minimize false positive results.

2^nd^ round – evolved α factors: Three evolved alpha factor pre-pro leaders previously described in papers were synthesized by GeneArt (Thermo Fisher Scientific) – PM1 laccase evolved alpha factor (PM1), *Pycnoporus cinnabarinus* laccase evolved alpha factor (PcL)^22,24^ and alpha factor evolved by K. Dane Wittrup group at MIT, Cambridge (Wit)^26^. The signal peptides were subjected to PCR reactions in order to obtain flanking regions homologous to the pYES2 vector at 5′ site and 4C1 laccase mutant at 3′ site. The 4C1 mutant was also subjected to PCR reaction in order to obtain a flanking region homologous with the signal peptides at 5′ and pYES2 vector at 3′ site. The PCR reactions were done with Q5 high fidelity 2x Master Mix (NEB) (Supplemental Table S11). The vector construction, transformation and cultivation were done as explained above. In this round of evolution, the screening was done differently than for other rounds of evolution. The plates were screened with ABTS assay at pH 3 and here only the expression levels were compared (by laccase activity in culture supernatant). The level of expression was also checked for a 100 ml flask format (30 °C, 220 rpm).

3^rd^ round – site saturation mutagenesis: In silico QM/MM MD simulations revealed the energy of interaction of each amino acid residue with laccase substrates. Those with energy higher than zero were analyzed as potential hot spots for mutagenesis. Parallelly, analysis of laccase–DMP complex model was done with HotSporWizard 2.0 software available at https://loschmidt.chemi.muni.cz/hotspotwizard/ according to the procedure explained before^53^. The functional hot spots given by the program were compared to QM/MM results. Two residues indicated by both methods were chosen for combinatorial mutagenesis studies – Arg 515 and Arg 561. The library was designed with the library design tool available at HSW 2.0 website, amino acid frequencies were chosen as the mode for selection of amino acids and minimal frequency was set to 5. The mutagenic PCR was conducted with Q5 high fidelity 2x Master Mix (Supplemental Table S12). The vector construction, transformation, cultivation and screening was performed in the same way as in the 1^st^ round of mutagenesis.

Production and purification: The colonies that exhibited the highest laccase activity were picked and cultivated in shaking flask format in SC Raffinose medium (galactose in MEM was exchanged with 2 g/L raffinose and no CuSO_4_ was added) for 72 h at 30 °C, 220 rpm. Next, the cultures were refreshed to OD_600_ 0.3 and cultivated in the same conditions for 6–8 h till OD_600_ reached 1. The main cultures were started with the addition of 10% pre-culture to the expression medium which contained 1 mM CuSO_4_. After 24 h of cultivation at 25 °C and 60 rpm, 10% galactose was supplemented and the cultivation was prolonged for the next 48 h. The cells were separated by centrifugation (10000 × g, 20 min, 4 °C) and filtration through 0.4 µm cellulose filters. Next, the culture supernatant was concentrated on 30 kDa Sartorius Hydrosart UF | MF Sartocon Slice 200 (Sartorius) using Sartorius tangential flow system followed by buffer exchanged to 20 mM Bis-Tris-HCl, pH 5.9 (buffer A for cation exchange chromatography). The conductivity of the sample was adjusted to buffer A and the sample was loaded to HiPrep QFF 16/60 column (GE Healthcare) connected to an Aktä system (GE Healthcare). The protein was eluted with 55% of buffer B (20 mM Bis-Tris-HCl with 200 mM NaCl, pH 5.9). Samples with laccase activity towards ABTS at pH 5 were pulled, concentrated on Sarticon with 10 kDa cut-off (Sartorius) with simultaneous buffer exchange to 50 mM Na-acetate, pH 4.4. The sample was loaded on two HiTrap SPFF 1 ml columns connected in a row (GE Healthcare). The protein was eluted with a linear gradient of 1 M NaCl. The fractions with laccase activity towards ABTS were pulled, concentrated on Sarticon with 10 kDa cut-off. In the last step of the purification protocol, the samples were subjected to gel filtration chromatography of Sephadex 200 column (GE Healthcare) in 50 mM Na-acetate buffer of pH 5 with 150 mM NaCl. The progress of the purification was monitored by SDS-PAGE electrophoresis on 12% TruPage precast gels (Sigma-Aldrich) stained with Pierce Silver Stain kit (Thermo Fisher Scientific).

Characterization: Laccase variants were concentrated on centrifugal filters with 10 kDa cut-off and subjected to characterization. The winner of the evolution was also characterized after purification. The determination of protein concentration was done according to the Bradford method with Bradford reagent (Sigma-Aldrich) according to the manufacturer’s instructions. The substrate specificity of laccases was tested towards ABTS (ε_420_ = 36000 M^−1^cm^−1^) and DMP (ε_469_ = 27500 M^−1^ cm^−1^). Appropriate laccase dilutions were prepared in such a way that 20 µl aliquots produced a linear response in the kinetic mode. pH stability was assessed for pH range from 2 to 8 (assessed after 24 h incubation at 4 °C), temperature stability from 20 to 80 °C (10 minutes incubation at a given temperature and 5 minutes incubation on ice), for both characteristics ABTS assay at pH 5 was used. The optimal temperature for the activity of laccase variants was assessed with ABTS, pH profiles were done for both substrates. The kinetic constants were determined for optimal pH towards ABTS and DMP. Reactions were conducted for 9 different substrate concentrations in 200 µl reaction volumes in McIlvaine buffer of optimal pH for the enzymes at 25 °C. Substrate oxidation was followed by measurement of absorption in kinetic mode. To calculate the values of Km and kcat, the average V_max_ was represented versus substrate concentration and fitted to a ligand binding fit with one-site saturation in SigmaPlot (version 11.0) software. Deglycosylation of the 4A9 mutant was achieved with PNGaseF (NEB) according to the manufacturer’s instructions.

Brown coal modification: The experiment studying the impact of laccase on brown coal modification was done with concentrated culture supernatants of Gr2, 4C1 and 4A9 laccases. Firstly, brown coal pretreated by ozonation^39^ was solubilized by *Gordonia alkanivorans* S7^5^. 200 µl of coal was mixed with 100 µl 100 mM DMP, 300 µl McIlvaine buffer pH 5. The reaction was started with the addition of 400 µl enzyme preparation of 1.7 U/ml. The control samples contained buffer instead of enzyme preparation. The 2 ml Eppendorf tubes with the mixtures were shaken with 150 rpm at 25 °C. 20 µl of samples were taken during 180 min of the process. The samples were diluted 10 times and absorbance at 450 nm and 650 nm were measured in order to monitor the release of humic and fulvic acids.

## Supplementary information

Supplementary Data.

## Data Availability

All data generated and/or analyzed during this study are included in this published article (and its Supplemental Information files) or are available from the corresponding author on reasonable request.
