# Supplementary material for: Evolved Fusarium oxysporum laccase expressed in Saccharomyces cerevisiae
Source: Sci Rep. 2020 Feb 24;10:3244. doi: 10.1038/s41598-020-60204-1 (PMC7039978; doi:10.1038/s41598-020-60204-1)
Supplement: Supplementary file 1 — Supplementary Data. [file 41598_2020_60204_MOESM1_ESM.pdf]

Scientific Reports

Supplemental Information for:

Evolved *Fusarium oxysporum* laccase expressed in *Saccharomyces cerevisiae*

Natalia Kwiatos,[a] Marzena Jędrzejczak-Krzepkowska,[a] Agnieszka Krzemińska,[b] Azar Delavari,[c] Piotr Paneth,[d] Stanisław Bielecki[a*]

[a] Institute of Technical Biochemistry, Faculty of Biotechnology and Food Sciences, Lodz University of Technology, Stefanowskiego 4/10, 90-924 Łódź, Poland
[b] Institute of Physics, Lodz University of Technology, Wólczańska 219, 90-924 Łódź
[c] Independent scholar
[d] Institute of Applied Radiation Chemistry, Faculty of Chemistry, Lodz University of Technology, Wróblewskiego 15, 93-590, Łódź, Poland

*stanislaw.bielecki@p.lodz.pl

Supplementary Materials

Results and Discussion

Molecular simulation studies. The Hatcher lignin model obtained the lowest binding energy estimation according to MOE, which may suggest that lignin itself is a great substrate for the laccase. However, literature data excluded the possibility of direct oxidation of lignin with laccase. Only a part of the compound enters the substrate binding pocket, but probably after oxidation, if such occurs, the molecule cannot leave the site causing enzyme inhibition[1,2]. Interestingly, the lowest scores were not obtained for any lignin derivatives, as it was suspected, but for quercetin and resveratrol – plant derived flavonoids. It opens a new route of studies for a different application. Polyphenol oxidases have been demonstrated before to react with flavonoids and influence their antioxidant activity[3]. What is more, laccases could be used in biosensor systems, for example for detection of a certain flavonoid[4].

Supplemental Table S1 The results of docking in MOE. The score is the estimation of the free energy of binding of the ligand from a given pose.

**Number of pose in moe**	**Substrate**	**Score**	**Residues show interaction**	**Formula**	**Molecular Weight**
21	Acetovanillone	-7.2945652	D293+H586	C9H10O3	166.17g/mol
47	Cafeic acid	-9.7197809	E589+H586	C9H8O4	180.16 g/mol
68	Catechol	-7.3656445	D293+H586	C6H6O2	110.11g/mol
145	4-hydroxyacetophenone	-6.5339026	E589+H586-153(D293+H586)	C8H8O2	136.15g/mol
170	4-hydroxybenzaldehyde	-6.5736947	D293+H586	C7H6O2	122.12g/mol
198	Acetosyringone	-8.002799	E589+H586	C10H12O4	196.2g/mol
255	Eugenol	-7.5420303	D293+H586	C10H12O2	164.2g/mol
309	Ferulic acid	-9.5593891	E589+H586	C10H10O4	194.18g/mol
338	Fulvic acid	-9.4501905	E589+H586-342(D293+H586+R515)	C14H12O8	308.24g/mol
351	Gallic acid	-10.128135	D293+H586	C7H6O5	170.12g/mol
399	Guaiacol	-7.4257646	D293+H586	C7H8O2	124.14g/mol
439	Guaiacyl4-o-5Guaiacyl	-9.6771402	D293+H586+F455	C16H18)4	274.0 g/mol
525	2,6-dimethoxyphenol	-7.2691011	D293+H586	C13H16O2	204.26g/mol
548	3,4-dihydroxybenzoicacid	-11.012486	D293+H586	C7H6O4	154.12g/mol
609	4-hydroxybenzoic acid	-7.7188001	D293+H586	C7H6O3	138.12g/mol
665	Methyl ferulate	-8.6468859	665(E589)-670(D293+H586)	C11H12O4	208.21g/mol
711	Methyl p-coumarate	-7.7609329	D293+H586	C10H10O3	178.18g/mol
746	Methyl sinapate	-9.1099777	E589+H519-763(D293+H586)	C12H14O5	238.24g/mol
790	Methyl syringate	-8.6711874	D293+H519	C10H12O5	212.2g/mol
827	Methyl vanilate	-8.0278959	E589+H586	$C_9H_{10}O_4$	182.17 g/mol
854	Phenol	-7.5222669	D293+H519	C6H6O	94.11g/mol
911	sinapic acid	-8.9225845	E589+H586	C11H12O5	224.21g/mol
922	Sinapyl alcohol	-9.3644028	R515+H586-928(E589+H586)-934(D293+H586)	C11H14O4	210.23g/mol
980	Syringaldehyde	-8.1325951	D293+H586	C9H10O4	182.17g/mol
1 075	Quercetin	-13.000735	D293+H586	C15H10O7	302.23g/mol
1 135	Resveratrol	-11.182361	E589+H586-1143(D293+H586+R561)	C14H12O3	228.24g/mol
1 190	Syringic acid	-9.7660789	D293+H586	C9H10O5	198.17g/mol

1 220	Vanillic acid	-9.4442663	D293+H586	C8H8O4	168.15g/mol
1 256	Vanilline	-8.9240456	D293+H586	C8H8O3	152.15g/mol
8	Pyrogallol	-9.0568705	D293+H586-57(E589+H586)	C6H6O3	126.11g/mol
14	p_coumaric acid	-6.6763859	E589+H586	$C_9H_8O_3$	164.0473 g/mol
80	Benzyloxybenzene	-8.2906733	F455+F436	C13H12O	184.23g/mol
34	Lignite	-15.797472	D293+H586		

Supplemental Table S2 HotSpotWizard results

Position	Residue	Mutability score	In tunnel	In catalytic pocket
**238**	Ser	9	yes	no
**324**	Leu	8	no	yes
**361**	Asp	8	no	yes
**398**	Lys	8	no	yes
**399**	Ala	8	no	yes
**435**	Thr	8	no	yes
**436**	Phe	8	yes	yes
**437**	Asp	8	no	yes
**511**	Gly	8	no	yes
**459**	Arg	7	no	yes
**515**	Arg	7	yes	yes
**517**	Gly	7	yes	yes
**561**	Arg	7	no	yes

Position	Residue	Mutability score	In tunnel	In catalytic pocket
**236**	Gln	6	yes	no
**292**	Phe	6	yes	no
**295**	Thr	6	yes	yes
**364**	Gly	6	yes	no
**434**	Val	6	no	yes
**458**	Gln	6	yes	yes
**509**	Ile	6	no	yes
**516**	Thr	6	yes	yes
**518**	Ala	6	yes	yes
**562**	Asn	6	no	yes
**589**	Glu	6	no	yes
**460**	Pro	6	no	yes

Classical culture optimization. Culture conditions were optimized for the production of recombinant laccase. Elevated concentration of copper sulfate and addition of galactose at the end of the first day of cultivation enhanced the detected activity in the culture supernatant. However, the most prominent change

was observed due to the decrease of shaking to only 60 rpm and the decrease of temperature to 25ºC (Supplemental Table S1). Lower temperatures were earlier reported to boost the expression of recombinant proteins [5,6]. Most literature data states that the optimal shaking conditions for recombinant *S. cerevisiae* cultures are around 220 rpm [7,8]. Vigorous shaking supplies the culture with oxygen which boosts the growth of the yeast, hence the amount of produced protein. In the case of GR2 laccase vigorous shaking causes a decrease in volumetric activity. This could be due to the production of a certain metabolite that inhibits or degrades the enzyme. Another hypothesis is that the enzyme is susceptible to the excess of oxygen caused by vigorous shaking leading to laccase degradation. Stationary conditions were not optimal because of the fact that the yeast grew very slowly, as well as the activity of laccase in culture broth (for 60 rpm the activity was twice higher than for 0 rpm). A similar situation was observed for growth at 20ºC (the activity was 3 times higher for 25ºC).

Supplemental Table S3 Optimization of culture conditions - improvement of activity in culture supernatant in a given conditions

**Conditions/Steps**	**1st step**	**2nd step**	**3rd step**	**4th step**	**5th step**
**$CuSO_4$ end concentration**	0.2 mM	1 mM	1 mM	1 mM	1 mM
**Shaking**	220 rpm	220 rpm	60 rpm	60 rpm	60 rpm
**Additional galactose**	-	-	-	+	+
**Temperature**	30ºC	30ºC	30ºC	30ºC	25ºC
**When maximal activity**	24 h	24 h	48 h	48 h	72 h
**Activity**	80-130 U/L	150 -200 U/L	250-300 U/L	350-400 U/L	500-600 U/L

Production, purification and characterization of 4A9 laccase variant. The SDS-PAGE analysis shows that the enzyme was purified nearly to homogeneity (Supplemental Figure S1). In order to estimate the size of the protein, the pure 4A9 mutant was subjected to deglycosylation with PNGaseF, which removes N-linked oligosaccharides from glycoproteins. It shows that oligosaccharides compose around 40% of laccase molecular weight (Supplemental Figure S2). A similar extent of glycosylation was noticed for other laccases, for example for *T. versicolor* laccase expressed in *S. cerevisiae* (60 kDa additional weight due to glycosylation)[5] or *B. alclada* laccase expressed in *P. pastoris* (27% of the total mass of the protein)[9]. According to the molecular mass calculated bioinformatically (72.4 kDa), 4A9 mutant should be positioned below the 75 kDa band, nonetheless, it is visible just above 75 kDa standard band (Supplemental Figure S2). The similar problem was noticed for *B. aclada* laccase and the author of the publication suggests that this may happen due to the

imprecision of SDS-PAGE analysis[9]. However, another reason may be the *O*-glycosylation as five possible *O*-glycosylation sites were found by NetOGlyc 4.0 server. Additional bands that appear for the deglycosylated protein may be due to the incomplete deglycosylation of part of the sample[5].

Supplemental Table S4 Purification of 4A9 mutant

	**Volume (ml)**	**Protein concentration (mg/ml)**	**Total protein (mg)**	**Activity (U/ml)**	**Specific activity (U/mg)**	**Total activity (U)**	**Yield (%)**
**Culture supernatant**	2000	0.02	40.00	0.15	7.5	300.0	100%
**Tangential flow filtration**	250	0.15	36.75	1.13	7.7	282.5	94%
**HiPrep QFF 16/60**	103	0.12	12.52	1.36	11.2	140.1	47%
**HiTrap SPFF**	2	1.88	3.76	36.6	19.5	84.0	28%
**Sephadex 200**	4	0,54	2,15	19	35.4	76.0	25%

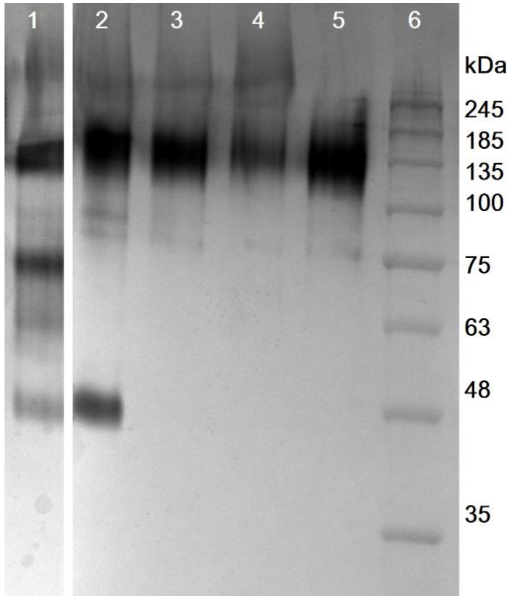

Supplemental Figure S1 SDS Page showing purification steps of 4A9 mutant. Line 1 – TFF, Line 2 – QFF, Line 3 – SPFF, Line 4 – Sephadex fraction 1, Line 5 – Sephadex fraction 2 Line 6 – MW marker. In each line 0.1 ug of protein was loaded. White space indicates that Line 1 was cut from another gel.

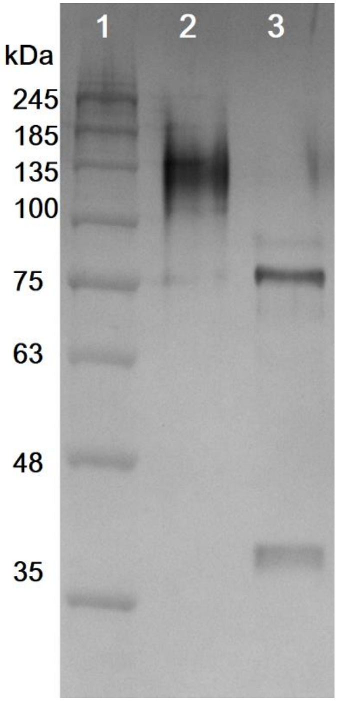

Supplemental Figure S2 Deglycosylation of 4A9 laccase. SDS PAGE analysis - Line 1: MW marker, Line 2: 4A9 laccase, Line 3: 4A9 laccase treated with PNGaseF, the band above 35 kDa corresponds to PNGaseF.

Materials and methods

Supplemental Table S5 Mass and van der Waals parameters introduced to the AMBER force field

Atom	Mass(u)	Van der Walss Radius [Å]
**Cu(I,II)**	63.546	1.17 from [10]

Supplemental Table S6 Equilibrium bond distances and stretching force constants introduced to the AMBER force field.

**Bond**	***kb* [kcal/(mol*Å^2)]**	***r*0(Å)**
**Cu1-S**	5.00	2.87 [11]
**Cu1-SH**	50.61	2.22
**Cu1-NB**	21.25	2.03
**Cu1-OH**	10.00	2.35

Supplemental Table S7 Equilibrium valance angles introduced to the AMBER force field.

**Angle**	**θ_0 (^{o})**
**Cu1-S -CT**	113.81
**Cu1-SH-CT**	101.31
**Cu1-NB-CR**	122.10
**Cu1-NB-CC**	122.90
**Cu1-NB-CV**	122.90
**Cu1-OH-H**	120.00
**S -Cu1-SH**	113.39
**S -Cu1-NB**	96.50
**SH-Cu1-NB**	122.50
**NB-Cu1-NB**	99.07
**OH-Cu1-NB**	90.00

Supplemental Table S8 Primers used in the study

**Amplification of:**	**Name**	**Sequence (5' → 3')**	**Tm (^{o}C)**
Vector construction	pYES_αfactor_fw	GACTCACTATAGGGAATATTAAGCTTGGTACCG AGCTATGAGATTTCCTTCAATTTTTACTG	67
Vector construction	αfactor-GR2Lac_rw	TCTCAATGGATGTTGGTAGGAATGATGATGATG ATGATGAGCTTCAGCCTCTCTTTTCT	70
Vector construction	αfactor_GR2Lac_fw	AGAAGAAGGGGTATCTCTCGAGAAAAGAGAGG CTGAAGCTCATCATCATCATCATCATTCCT	71
Vector construction	GR2Lac_pYES_rw	CATGATGCGGCCCTCTAGATGCATGCTCGAGCG GCCTTAAATACCAGAGTCACCTTCGAA	75
Laccase gene 1st round of	FRpYES	GCAGCTGTAATACGACTCAC	52

mutagenesis; Fragment I, 3rd round of mutagenesis			
Laccase gene 1st round of mutagenesis; Fragment III, 3rd round of mutagenesis	RWpYES	GACATAACTAATTACATGATGCGG	52
PcL and PM1 signal peptide	PcL_RW	GAGTGTGCGGGTGGTTTGGTCTCAATGGATGTT GGTAGGATCTTTTCTCGAGAGATACCC	60
PcL signal peptide (with STE13 site)	PcL_RW_STE	GAGTGTGCGGGTGGTTTGGTCTCAATGGATGTT GGTAGGAAGCTTCAGCCCCTCTTTT	64
PM1 signal peptide (with STE13 site)	PM1_RW_STE	GAGTGTGCGGGTGGTTTGGTCTCAATGGATGTT GGTAGGAAGCTTCAGTCTCTCTTTTCT	60
Wit signal peptidw	Wit_RW	GAGTGTGCGGGTGGTTTGGTCTCAATGGATGTT GGTAGGATCTTTTATCGAGTTGTACCC	58
PM1, PcL, Wit all with or without STE13 site	pYES_LF_FW	GACTCACTATAGGGAATATTAAGCTTGGTACCG AGCTATGAGATTTCCTTCAATTTTTACTG	59
4C1 mutant with flanking regions homologous with PcL and PM1signal petide	LAC_PcL_FW	CATTGCTGCTAAAGAAGAAGGGGTATCTCTCGA GAAAAGATCCTACCAACATCCATTGAG	61
4C1 mutant with flanking regions homologous with PcL signal petide with STE13 site	LAC_PcL_STE_FW	AGAAGAAGGGGTATCTCTCGAGAAAAGAGGGG CTGAAGCTTCCTACCAACATCCATTGAG	61
4C1 mutant with flanking regions homologous with PM1 signal petide with STE13 site	LAC_PM1_STE_FW	AGAAGAAGGGGTATCTCTCGAGAAAAGAGAGA CTGAAGCTTCCTACCAACATCCATTGAG	61
4C1 mutant with flanking regions homologous with Wit signal peptide	LAC_Wit_FW	CATTGCTGCTAAAGAAGAAGGGGTACAACTCGA TAAAAGATCCTACCAACATCCATTGAG	61
4C1 mutant	LAC_pYES_RW	GGCCGCTCGAGCATGCATCTAGAGGGCCGCATC ATGTTAAATACCAGAGTCACCTTCGAAG	64
Fragment II, 3rd round of mutagenesis	Fw515	TCGAGGGTATCTCCGCTVBAACTGGTGCTCA	77
Fragment I, 3rd round of mutagenesis	Rw515	GAATAGGATGAGCACCAGTTVBAGC	67
Fragment III, 3rd round of mutagenesis	Fw561	ATGTTGTTTTGTTGCCARVVAACG	65
Fragment II, 3rd round of mutagenesis	Rw561	GCGATAACAACGAAACCGTTBBYTGGCAACA	73

The PCR conditions in which 1-3 mutations were introduced into the coding sequence of GR2 laccase were determined. The PCRs of set DNA concentration and the number of cycles were done with changing $MnCl_2$ concentration (0.025 mM, 0.05 mM, 0.1 mM) (Supplemental Table S7). The PCR product obtained with 0.025 mM MnCl2 was used for the target PCR reaction.

Supplemental Table S9 PCR conditions for setting the $MnCl_2$ concentration for error-prone PCR

Component	Volume (µl)
10 x Pol Buffer B (Eurx)	5
10 ng/ µl pYES_GR2Lac	0.5
5 mM dNTP	1
25 µM FRpYES primer	1
25 µM RWpYES primer	1
1 mM $MnCl_2$	1.25
Color Taq Polymerase (Eurx), 1u/µl	1.25/2.5/5 µl
Nuclease free water	up to 50 µl

Temperature	Time
95ºC	5 min
95ºC	30 s
54ºC	45 s
72ºC	2 min
35 cycles	
72ºC	7 min
4ºC	∞

Table S10 PCR conditions for the first generation of mutagenesis

Component	Volume (µl)
10 x Pol Buffer B (Eurx)	5
10 ng/ µl pYES_GR2Lac	0.5
5 mM dNTP	1
25 µM FRpYES primer	1
25 µM RWpYES primer	1
1 mM $MnCl_2$	1.25
Color Taq Polymerase (Eurx), 1u/µl	1.25 µl
Nuclease free water	up to 50 µl

Temperature	Time
95ºC	5 min
95ºC	30 s
54ºC	45 s
72ºC	2 min
35 cycles	
72ºC	7 min
4ºC	∞

Table S11 PCR conditions for the second generation of mutagenesis

Reagent	Volume (µl)
Q5 High-Fidelity 2x Master Mix	25
10 mM Forward Primer	2.5
10 mM Reversed Primer	2.5
pYES LF4C1/evolved alpha factor	0.5
Nuclease free water	19.5

Temperature	Time
98ºC	30 s
98ºC	10 s
60/62ºC	30 s
72ºC	30 s/kb
35 cycles	
72ºC	2 min
4ºC	∞

Table S12 PCR conditions for the third generation of mutagenesis

Component	Volume (µl)	Temperature	Time
Q5 High-Fidelity 2x Master Mix	25	98ºC	30 s
10 mM Forward Primer	2.5	98ºC	10 s
10 mM Reversed Primer	2.5	Fragment I: 64ºC<br>Fragment II: 72ºC<br>Fragment III: 62ºC	30 s
pYES wit4C1	0.5	72ºC	Fragment I: 1 min<br>Fragment II: 6 s<br>Fragment III: 10 s
Nuclease free water	up to 50 µl	35 cycles	
		72ºC	2 min
		4ºC	∞

Classical culture optimization

Having performed the second round of evolution, culture conditions in shaking flasks were optimized. In the first step, the concentration of $CuSO_4$ in the culture medium was optimized. The recombinant yeast was cultivated in 20 ml MEM medium in 100 ml shaking flasks. The following end concentrations of copper sulfate were applied: 0.2, 0.5, 1, 2, 4 mM. Each condition was checked in triplicate. The next step was to optimized shaking – 220 rpm, 100 rpm, 60 rpm and no shaking was checked; each condition was applied to three cultivations in 100 ml flasks. The third step was supplementation of culture with

galactose. Three cultures were run without changes and the next three were supplemented with 10% end concentration of galactose. The last step of optimization was establishing the most adequate temperature for protein expression. 30ºC, 25ºC and 20ºC were tested.

Supplemental References

1. Maijala, P. *et al.* Action of fungal laccases on lignin model compounds in organic solvents. *J Mol Catal B Enzym* **76**, 59–67 (2012).
2. Munk, L., Sitarz, A. K., Kalyani, D. C., Mikkelsen, J. D. & Meyer, A. S. Can laccases catalyze bond cleavage in lignin? *Biotechnol. Adv.* **33**, 13–24 (2015).
3. Riebel, M. *et al.* Antioxidant capacity of phenolic compounds on human cell lines as affected by grape-tyrosinase and Botrytis-laccase oxidation. *Food Chem* **229**, 779–789 (2017).
4. Gomes, S. a. S. S., Nogueira, J. M. F. & Rebelo, M. J. F. An amperometric biosensor for polyphenolic compounds in red wine. *Biosens Bioelectron* **20**, 1211–1216 (2004).
5. Iimura, Y., Sonoki, T. & Habe, H. Heterologous expression of Trametes versicolor laccase in Saccharomyces cerevisiae. *Protein Expr. Purif.* **141**, 39–43 (2018).
6. Viña-Gonzalez, J., Elbl, K., Ponte, X., Valero, F. & Alcalde, M. Functional expression of aryl-alcohol oxidase in Saccharomyces cerevisiae and Pichia pastoris by directed evolution. *Biotechnol. Bioeng.* **115**, 1666–1674 (2018).
7. Pardo, I., Vicente, A. I., Mate, D. M., Alcalde, M. & Camarero, S. Development of chimeric laccases by directed evolution. *Biotechnol. Bioeng.* **109**, 2978–2986 (2012).
8. Mateljak, I., Tron, T. & Alcalde, M. Evolved α-factor prepro-leaders for directed laccase evolution in Saccharomyces cerevisiae. *Microb Biotechnol* **10**, 1830–1836 (2017).
9. Kittl, R. *et al.* A chloride tolerant laccase from the plant pathogen ascomycete Botrytis aclada expressed at high levels in Pichia pastoris. *J. Biotechnol.* **157**, 304–314 (2012).
10. Bartolotti, L. J., Pedersen, L. G. & Charifson, P. S. Long range nonbonded attractive constants for some charged atoms. *J. Comput. Chem.* **12**, 1125–1128 (1991).
11. De Kerpel, J. O. A. & Ryde, U. Protein strain in blue copper proteins studied by free energy perturbations. *Proteins* **36**, 157–174 (1999).
